# Reduced Discrimination in the Tritanopic Confusion Line for Congenital Color Deficiency Adults

**DOI:** 10.3389/fpsyg.2016.00429

**Published:** 2016-03-30

**Authors:** Marcelo F. Costa, Paulo R. K. Goulart, Mirella T. S. Barboni, Dora F. Ventura

**Affiliations:** ^1^Laboratório de Psicofisiologia Sensorial, Departamento de Psicologia Experimental, Instituto de Psicologia, Universidade de São PauloSão Paulo, Brasil; ^2^Núcleo de Neurociências e Comportamento e Núcleo de Neurociências Aplicadas, Universidade de São PauloSão Paulo, Brasil; ^3^Núcleo de Teoria e Pesquisa do Comportamento, Universidade Federal do ParáBelém, Brasil

**Keywords:** chromaticity discrimination, anomalus trichromacy, chromatic thresholds, color vision, tritanopic color confusion line, visual psychophysics

## Abstract

In congenital color blindness the red–green discrimination is impaired resulting in an increased confusion between those colors with yellow. Our post-receptoral physiological mechanisms are organized in two pathways for color perception, a red–green (protanopic and deuteranopic) and a blue–yellow (tritanopic). We argue that the discrimination losses in the yellow area in congenital color vision deficiency subjects could generate a subtle loss of discriminability in the tritanopic channel considering discrepancies with yellow perception. We measured color discrimination thresholds for blue and yellow of tritanopic channel in congenital color deficiency subjects. Chromaticity thresholds were measured around a white background (0.1977 u′, 0.4689 v′ in the CIE 1976) consisting of a blue–white and white–yellow thresholds in a tritanopic color confusion line of 21 congenital colorblindness subjects (mean age = 27.7; *SD* = 5.6 years; 14 deuteranomalous and 7 protanomalous) and of 82 (mean age = 25.1; *SD* = 3.7 years) normal color vision subjects. Significant increase in the whole tritanopic axis was found for both deuteranomalous and protanomalous subjects compared to controls for the blue–white (*F*_2,100_ = 18.80; *p* < 0.0001) and white–yellow (*F*_2,100_ = 22.10; *p* < 0.0001) thresholds. A Principal Component Analysis (PCA) found a weighting toward to the yellow thresholds induced by deuteranomalous subjects. In conclusion, the discrimination in the tritanopic color confusion axis is significantly reduced in congenital color vision deficiency compared to normal subjects. Since yellow discrimination was impaired the balance of the blue–yellow channels is impaired justifying the increased thresholds found for blue–white discrimination. The weighting toward the yellow region of the color space with the deuteranomalous contributing to that perceptual distortion is discussed in terms of physiological mechanisms.

## Introduction

Congenital color blindness is a genetic condition in which male subjects show impairment in performing red–green discriminations increasing the confusion between those colors with yellow. The chromatic discrimination impairment can vary from a very weak loss of discrimination mediated by the L-cone or M-cone – anomalous trichromacy – to total loss of discrimination – dichromacy (Motulsky, 1988; Neitz and Neitz, 1995; Neitz et al., 2004; Deeb, 2005). Two post-receptoral channels exclusively carrying L- and M-cone information building physiologically opponent center-surround input processing in which signals from the L- and M-cones are antagonists – often called red–green opponency (De Valois and Abramov, 1966; De Valois and Jacobs, 1968; Lee et al., 1989; Lee, 1996). The Parvocellular (PC) pathway is suggested to carry the color information to the visual cortex and the Magnocellular (MC) pathway the luminance information (Lee, 1996).

**FIGURE 1:**
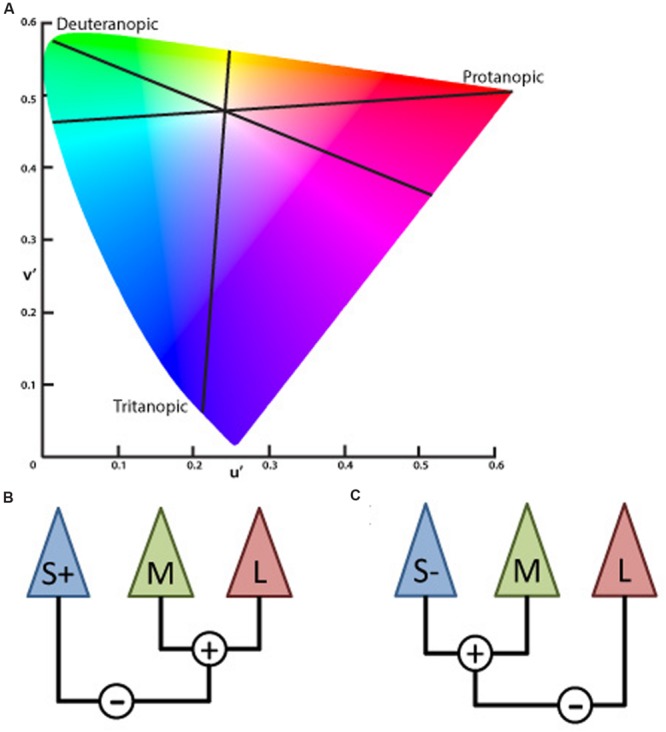
**(A)** Graphic representation adapted from the CIE 1976 L^∗^u^∗^v^∗^ Color Space and u′, v′ Uniform Chromaticity Scale Diagram (www.cie.co.at/ index.php/Publications/Standards). This spatial representation of colors is a non-linear transformation from the CIE 1931 L^∗^x^∗^y^∗^ color space to attempt a perceptual uniformity. The black lines represent the three – protanopic (L-cone), deuteranopic (M-cone), and tritanopic (S-cone) – color confusion axis in which colors of both sides along each of those lines could be mixed to be confused with the white center. **(B)** Schematic view of the S, M, and L cones contribution to the chromatic information of the koniocellular pathway, based on the physiological recordings of cone inputs to the lateral geniculate nucleus (LGN) of *Macaca fascicularis* performed by Tailby et al. (2008). Cone input configuration of the S+ cells in which the S-cone signal is opposed to the sum of the L- and M-cones. **(C)** Cone input of the S- cells in which the S-cone signal is summed with the M- cone and opposed to the L- cone.

The inputs of the L- and M-cones also contribute to another post-receptoral channel in which the S-cone signal is processed opponent to the L and M-cone signals combined – subjects show impairment in performing blue–yellow color discriminations increasing the confusion between those colors with white – called blue–yellow opponency, projecting to visual cortex via Koniocellular (KC) pathway of the LGN (Silveira et al., 1999; Teufel and Wehrhahn, 2000; Szmajda et al., 2006; Roy et al., 2009; Packer et al., 2010). The lines on the CIE color diagram corresponding to the red–green (protan and deutan) and blue–yellow (tritanopic) color opponency are the color confusion axes (**Figure 1**).

A recent study characterizing the S+ and S- cells of the macaque LGN showed critical asymmetries between those S-cone cells (Tailby et al., 2008). Three main results were found in that study. The prevalence of S+ and S- cells had similar proportions which differ from previous studies reporting higher proportion of S+ cells. The chromatic proprieties of the S+ and S- cells show differences in the anatomical level. S- cones sum information over a larger retinal area than the S+ cones. Additionally, the input from L and M cones linked to the S+ cones had the same sign (summed). Differently from the S-cones in which the M cone has the same sign than the S-cone (summed) and the L cone had an opposite sign.

Considering the post-receptoral contributions of L and M cones in the blue–yellow opponency channel, the proportions of the L and M cells directed by ON or OFF bipolar cells should be the basis for different chromatic configuration of the S+ and S- cells. New electrophysiological evidences in the macaque’s LGN shows that, given the S+ cells receive inputs from L and M cones over a larger region of the retina, there were more chances of the L and M inputs having the same sign [S against (L + M)] (**Figure 1B**). Since S- receives inputs from smaller retina areas, and the proportion of L cones are higher than M cones, there is a higher chance of the inputs that come from the L and M cells shows opponent signs that configure the channel [(S- +M) against L] (Tailby et al., 2008; **Figure 1C**).

The authors also found a strong alignment to tritanopic axis for the physiological responses recorded in S+ cells, unlike the S- cells in which responses recorded generates a color confusion axis varying between the S-cone axis and the L - M axis. This means that the S- cells could be much more affected by the spectral sensitivity changes in the M or L cones. Considering the S- cells more variable responses behavior we argue that the changes in the spectral sensitivity of L or M cones, such as those characteristic of congenital anomalous trichromats, will probably impact the tritanopic confusion axis.

Based on that physiological information, we could suppose that in deuteranomalous and in protanomalous subjects the spectral sensitivity shifts would affect more the responses of S- cells. In addition, the S- cells show lower contrast sensitivity and stronger habituation susceptibility. The S+ cells discrimination would be less affected by spectral sensitivity changes of L and M cones since they show more restricted discrimination to tritanopic axis in both deutanomalous and protanomalous subjects. Thus, for the S- cells pathway we expect a more drastic effect in discrimination since this subtype is less specific to the tritanopic axis.

Behavioral studies of chromatic discrimination on the tritanopic color confusion axis in congenital color blindness are nevertheless scarce. Tritanopic discrimination was studied in patients with glaucoma, using Farnsworth 100-hue and the Besacon anomalometer. The results showed a significant shift to the blue part of the anomalous coefficient at the anomalometer equation and a higher total error score in the FMH100 with a lower discrimination at the blue region caps suggesting impairment in the S-cone pathway (Huang et al., 1993; Mantyjarvi and Tuppurainen, 1994). That result also could support the findings of another study showing a greater spectral shift in the blue region than in the yellow region of the tritanopic color confusion axis (De Valois et al., 1997). However, the whole picture is not too clear. Under high light levels the S-cone pathway could mediate some red–green discrimination in dichromatic subjects (Mcmahon and MacLeod, 1998).

In this paper, we investigate the chromaticity discrimination in the tritanopic color confusion axis in anomalous trichromats and dichromats subjects, assuming that their impaired L or M cone spectral sensitivity should impair their tritanopic discrimination. Our hypotheses are: (1) Protanomalous and deuteranomalous subjects have similar tritanopic discrimination as normal color vision subjects since on a S+ channel the restricted responses to S cone axis would preserve the discrimination despite the yellow region shifts to more reddish or greenish regions away from the axis, respectively; (2) Protanomalous and deuteranomalous subjects exhibit different from normal color discrimination on tritanopic axis since the S- cells had increased sensitivity to spectral shifts. Both hypotheses are possible since the proportion of S+ and S- pathways were found similar in the macaque LGN (Tailby et al., 2008).

## Materials and Methods

### Subjects

We evaluated 82 subjects with normal color vision recruited among the Institute of Psychology of the University of São Paulo students and staff with mean age of 25.1 years (*SD* = 3.56, 40 males). Anomalous trichromats (*n* = 21, mean age 27.7, *SD* = 5.6, all male) were also from the University of São Paulo students and staff. Demographic data is presented in **Table 1**. All subjects underwent a complete ophthalmological examination, including best-corrected visual acuity measurement, slit-lamp biomicroscopy and optic disk evaluation with the pupils dilated, with a 78-diopter lens. Inclusion criteria were best-corrected visual acuity of 20/20 or better measured monocularly at 4 m using an ETDRS chart – tumbling E (Xenonio, Sao Paulo, Brazil), refraction of ≤3.0 diopters considering the spherical equivalent of astigmatism values, absence of ophthalmological diseases and absence of known neurological and systemic diseases.

**Table 1:** Demographic data of the controls.

Age	CCT trivector			CCT ellipse			Anomalous quotient	Matching point (amplitude in anomaloscope units)	CCT^†^	Anomalous^%^
	Protan	Deutan	Tritan	Length	Axis	Angle				
**Controls**										
25.0	36.3	36.5	56.1	0.0103	1.45	76.3	0.97	37 (1.0)		
(3.6)^∗^	(11.0)	(10.1)	(15.9)	(0.003)	(0.25)	(17.8)	(0.10)			
**Anomalous trichromats**								
23	315	312	141	0.0474	1.86	170.2	1.56	45 (11.0)	PN	PN
32	907	131	46	29.267	309.55	4.6	1.47	44 (19.0)	PN	PN
22	640	251	62	0.1658	9.11	3.5	1.61	45.5 (20.0)	PN	PN
34	268	1100	85	0.1391	10.49	167.0	0.83	33.5 (17.5)	DT	DN
25	265	332	85	0.0973	9.13	5.1	0.85	34 (15.0)	DN	DN
36	265	1100	96	0.2311	9.42	168.0	0.72	31 (20.0)	DT	DN
35	1100	261	70	0.1745	11.45	2.6	1.39	43 (19.0)	PT	PN
24	240	1100	80	132.882	1266.18	166.7	0.55	26.5 (68.0)	DT	DN
25	156	642	70	0.0324	2.98	167.5	0.80	33 (13.5)	DN	DN
27	33	140	88	0.583	4.36	169.1	0.85	34 (23.0)	DN	DN
25	216	638	78	0.0384	4.1	174.8	0.85	34 (19.5)	DN	DN
30	284	1100	83	0.0286	3.55	177.7	0.60	28 (23.5)	DT	DN
39	392	1100	129	0.2614	12.41	167.8	0.57	27 (12.5)	DT	DN
38	255	908	79	0.0949	4.45	172.8	0.62	28.5 (22.0)	DN	DN
23	69	131	70	0.0216	2.22	173.5	0.79	32 (13.5)	DN	DN
23	322	218	63	0.0451	4.7	4.3	1.48	44 (16.5)	PN	DN
26	319	845	102	0.1634	9.86	160.7	0.89	35 (19.5)	DN	DN
25	1006	354	109	0.2152	12.8	4.00	1.66	46 (12.5)	PN	PN
22	639	250	65	0 1657	9.12	3.9	1.81	47.5 (16.5)	PN	PN
23	310	316	139	0.0476	2.06	173.3	0.90	35 (13.0)	DN	DN
25	210	642	75	0.0383	4.5	176.2	0.66	29.5 (24.0)	DN	DN

*Dlchromat and Anomalous Trichromat Subjects.*

*^†^Diagnosis Based on CCT Trivector; ^∗^standard deviation.*

*^%^Diagnosis based on Anomalous cope.*

*PT, Protanope/PN, Protanomalous; DT, Deuteranope/DN, deuteranomalous.*

The study was approved by the Ethics Committee of the Institute of Psychology, University of Sao Paulo, and all subjects gave a signed informed consent to participate in the experiment. All were unaware to the specific experimental question. This study is also in accordance with the ethical standards laid out in the 1964 Declaration of Helsinki.

### Equipment and Procedures

The evaluation of the color discrimination was performed using the commercial version of the Cambridge Color Test, CCT (v2.0 [Cambridge Research Instruments]) installed in a personal computer (Dell Dimension XTC-600) with a graphic card VSG 2/5 (Cambridge Research Instruments). The stimuli were generated in a high-resolution color monitor, Sony FD Trinitron model GDM-F500T9 (Sony). Testing was conducted in a dark room with the patients positioned 3 m away from the screen.

**FIGURE 2:**
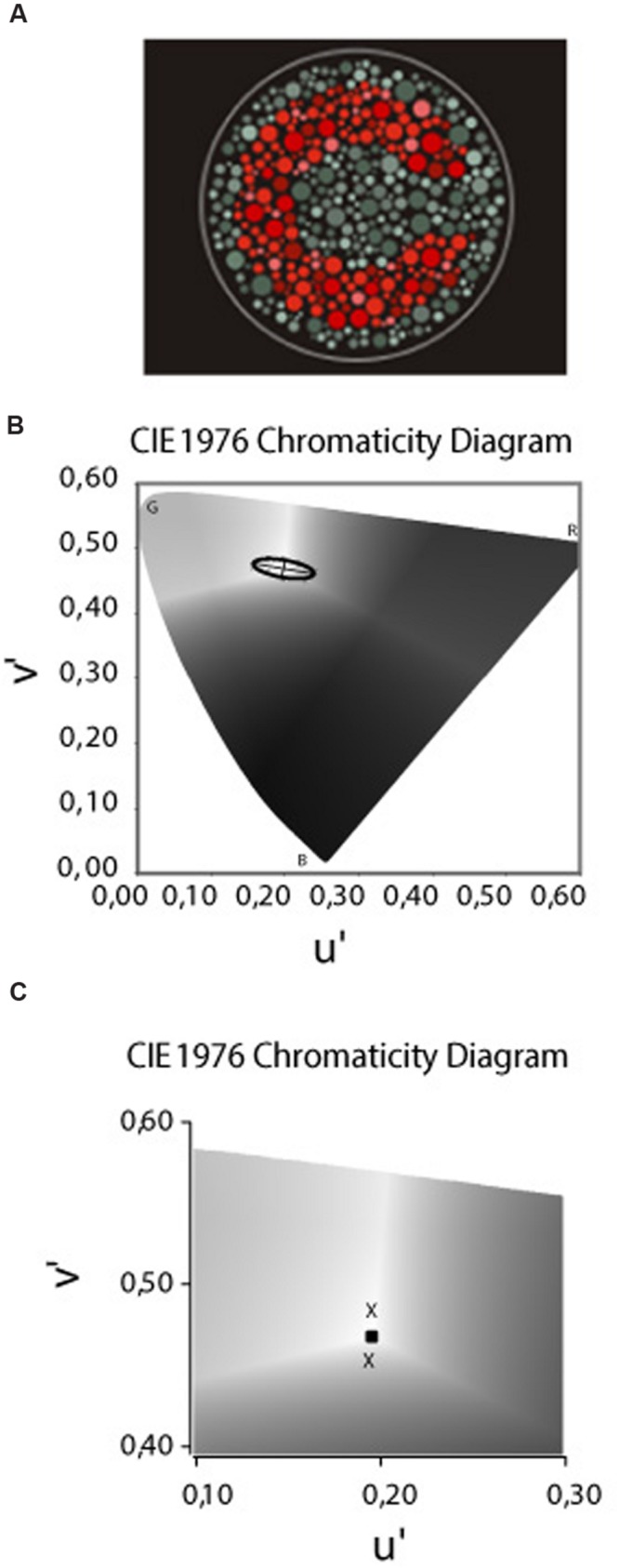
**(A)** The target – a Landolt “C” – that differed in chromaticity from the single neutral background [coordinates 0.1977, 0.4689 of the (CIE) u′v′ 1976 color space]. The target and background composed of small patches of varying sizes and luminance levels randomly distributed in the display. **(B)** The CIE 1976 u′ v′ Chromaticity Diagram used to draw the MacAdam ellipses or the non-discriminable area. The letters B, G, and R represent the spatial position of the blue, green, and red primaries, respectivelly. The bright central area (coordenates 0.1977 u′, 0.4689 v′) was the stimulus background and the “Xs” represents the thresholds measured in the each of the eight color confusion vectors. The ellipses drawn is regarding to one of our deuteranomalous subjects based on the method of the least squares. **(C)** Magnification of the Chromaticity Diagram in which we present a sample of thresholds measured in the tritanopic axes to the yellow (upper cross) and blue (lower cross) directions. The central square represents the backgroud chromaticity coordinates.

The stimulus provided by the CCT was similar to those used in the pseudoisochromatic plate tests, such as the Ishihara test (Kanehara Traiding Co.) or the AO H-R-R (v.4 Richmond Products, Albuquerque, NM, USA 2006). The target consisted of a Landolt “C” that differed in chromaticity from the single neutral background [coordinates 0.1977, 0.4689 of the International Commission on Illumination (CIE) u′v′ 1976 color space] (**Figure 2A**). The size of the Landolt C gap corresponded to 1.25° of visual angle, with the outer diameter 5.40° and the inner diameter 2.75° at the test distance of 3 m. Both the target and the background were composed of small patches of varying sizes (0.5–2 cm in diameter) and six luminance levels [8, 10, 12, 14, 16, and 18 candela (cd)/m2] randomly distributed on the screen. This visual layout creates spatial and luminance noise aiming to avoid the influence of cues derived from luminance differences and/or simultaneous contrast from target contours in the intended hue discrimination.

The target was randomly presented with its opening in one of four positions: up, bottom, right, and left (4-Alternative Forced Choice strategy). The patient’s task was to press one of the four buttons of the response box [CT3 (Cambridge Research Instruments)], to indicate the position of the “C” opening. The patients had up to 5 s to give the response. A psychophysical staircase procedure was used for threshold determination. Each staircase began with a saturated chromaticity (strong color very different from the background), which was changed along the vector connecting it to the background chromaticity. The change depended on the patient’s response: the target chromaticity approached the background chromaticity every time there was a correct response and moved away from it every time there was an incorrect response or no response. The chromaticity excursion along the vectors ranged from 0.1100 to 0.0020 u′v′ units of CIE 1976 color space. After six staircase reversals, the program automatically calculated the threshold for that vector as the average of the chromaticities corresponding to the last four reversals. The step size used in the staircase followed a dynamic rule; basically the size considered in the performance of the last responses to calculate the next values to present [for more details on the CCT methodology, see the work of (Regan et al., 1994) and, for CCT norms, see the work of (Ventura et al., 2002, 2003b; Costa et al., 2006; Paramei, 2012)].

In order to measure the thresholds along the tritanopic color confusion line, we used the CCT protocol for the construction of a discrimination ellipse (MacAdam ellipse) for the type of color impairment and their respective severity classification (**Figure 2B**). However, some methodological adjustments were performed since we were measuring only one of eight axes. The ellipses adjustment is performed using the Least Squares Method and our empirical experiment reveal that in many congenital color deficit subjects the ellipses fitting generate distortions in the tritanopic axes in which the ellipses contour passes away from the threshold measured. The distortion in the tritanopic axis, probably, occurs in the congenital color deficient subjects since, different from the subjects with normal color vision, they had significantly worse thresholds in the red–green axes compared to the blue–yellow axis which are perpendicular. The ellipses adjustment considers the Laplace distribution of the error which uses a symmetric two-sided exponential distribution to model the error distribution, and the sum of absolute deviation as estimation error; in our case, applied in an asymmetrical distribution of the thresholds data around background.

Since we were exclusively interested in the tritanopic discrimination, and to avoid the distortions in the ellipses fitting, as previously described, we considered the value of the thresholds measured at 90° and at 270° directions from the background described in u′ v′ coordinates of the CIE 1976 color space, and not the ellipses fitting to that color confusion axis (**Figure 2C**).

The staircases corresponding to these vectors were run in interleaved pairs randomly chosen by the software. We used the thresholds measured in the tritanopic axes as an indicator of the subject’s performance in color discrimination for “yellow” and “blue” sides against the white background.

### Statistical Analysis

Statistical analysis was performed with the software Statistica (v.12 StatSoft Inc. CA, USA, 2012). A full descriptive analysis was performed. Statistical differences among the groups were verified with the One-Way ANOVA. We use the Fisher least square differences (LSD) *post hoc* comparison, to determine the significant differences between group means in the ANOVA test. We used the method of PCA, for the purpose of variables diagnostics, computing factor coordinates and factor score coefficients as a classification technique, highlighting the relations among variables (thresholds for the blue–white and white–yellow directions of the tritanopic color confusion line in subjects with protan, deutan color vision defects and normal color vision) and cases (subjects). Adhesion to the normal distribution was checked with the Kolmogorov–Smirnov test.

## Results

**FIGURE 3:**
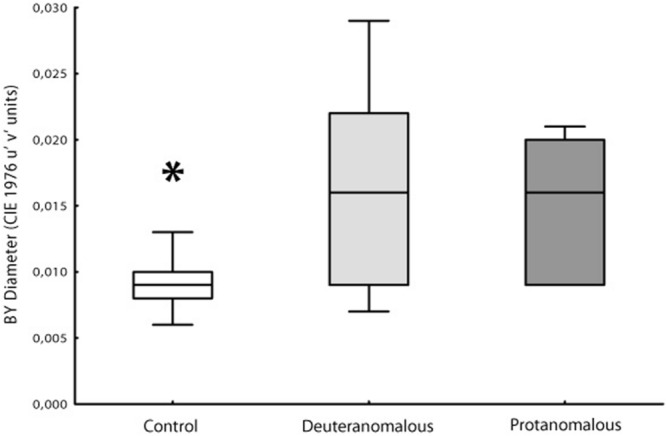
**Box Plot showing the median and interqurtile ranges with brackets showing the 95% of blue–yellow discrimination diameter.** Normal color vision subjects had significant better discrimination than deuteranomalous or protanomalous subjects. Control’s are statistically significant better than others (^∗^*p* > 0.001).

Thresholds measurements were performed in both control and congenital color vision impairment groups. The mean value obtained for the tritanopic axis was 0.0096 u′v′ units (*SD* = 0.00244) for controls and 0.0160 u′v′ units (*SD* = 0.0070) for the congenital color impairment group. The longer diameters measured in the color congenital impairment group had a statistical difference (*F*_2,100_ = 46.95; *p* < 0.0001) compared with those of the Control group. The breakdown analysis performed in the congenital color impairment group tested for differences between the subjects with protan (mean diameter 0.0150 u′v′ units; *SD* = 0.0055) and deutan (0.0166 u′v′ units; *SD* = 0.0078) color defects. No statistical differences were found between these two groups, but both of them differ statistically from the control group subjects (*F*_1,101_ = 25.32; *p* < 0.0001; **Figure 3**).

**FIGURE 4:**
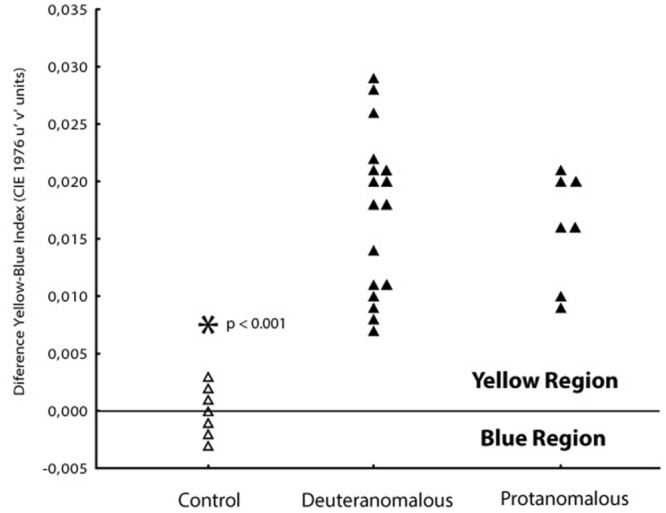
**Differences between thresholds obtained at 90 and 270° axes in order to analyses the yellow–blue symmetry.** Normal color vision subjects (△) had thresholds spread symmetrically between the orthogonal axes with a mean blue–yellow index around zero. Both protanomalous and deuteranomalous subjects (▲) had a asymmetrical shift toward the yellow direction (positive values) showing worse discrimination to that direction.

In order to investigate if the increase in tritanopic thresholds was symmetric to blue and yellow regions of the color space, we calculate a blue–yellow difference index, in which for each subject we subtracted the thresholds of blue from the yellow. Values around zero suggest symmetry between those thresholds at the color space. Positive values suggest higher thresholds for the yellow region and negative values suggest higher thresholds toward the blue region of the tritanopic axis (**Figure 4**). Statistical differences were found for protan (mean difference 0.0150 u′v′ units; *SD* = 0.0014) and deutan (0.0165 u′v′ units; *SD* = 0.0048) color defect subjects with normal color vision subjects (-0.0005 u′v′ units; *SD* = 0.0015; *F*_1,101_ = 199.71; *p* < 0.0001) for both blue and yellow distances.

**FIGURE 5:**
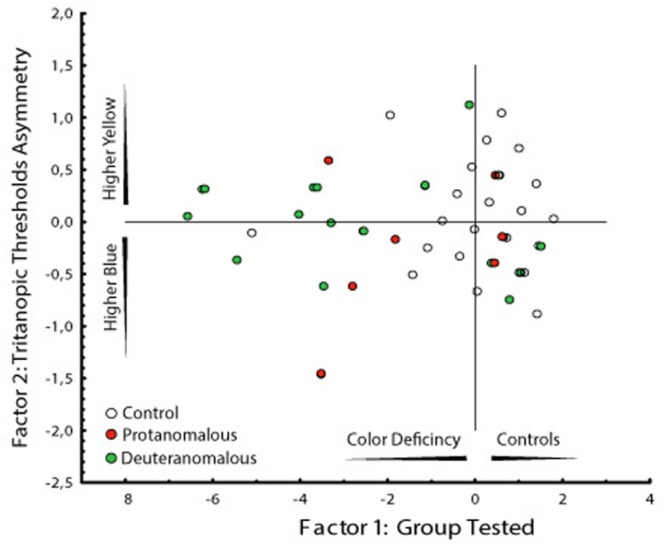
**Graphical analysis of the two factors computed by the PCA.** Factor 1 represent the subject groups (controls and congenital color deficiency) and factor 2 the thresholds to the yellow and blue regions of the tritanopic axes. It is interesting that the deuteranomalous subjects (labeled 3) had longer distances from the central than protanomalous (labeled 2) and controls (labeled 1), which means that they were the more affected group (more to the left side) and they showed the worse yellow color discrimination (more to upper side).

Since the higher dispersion of the thresholds values in color congenital deficiency subjects could be masking possible weighting for yellow or blue regions of the tritanopic axis, we performed a PCA to investigate if there were asymmetries supporting differences in those axis’ regions. As we could see, we found a statistically significant discrimination between the blue and yellow thresholds related to the total distance in which the yellow distance is an isolated component suggesting it as a more affected region of the tritanopic axis. The PCA results are shown in **Table 2** and **Figure 5**.

**Table 2:** Principal component analysis (PCA) factors.

	Factor 1	Factor 2
BY yellow region	-0,944736	0,327832
BY blue region	-0,954880	-0,296992
BY diameter	-0,999658	-0.026132

Two factors were obtained in the PCA analysis. The first factor represents the subject groups (controls and congenital color deficiency), and the second factor represents the thresholds of the yellow and blue regions of the tritanopic axis. In **Table 2** we can see a high degree of similarity, based on correlation analysis, suggesting a strong association between the variables for the first factor (control vs. color deficient). However, the second factor (blue vs. yellow thresholds in the tritanopic axis) shows a different projection of the individual cases of congenital color deficiency subjects to the yellow region of the chromaticity diagram, when compared with the blue region. Negative values of the first factor indicate a strong effect of color deficiency subjects and the second factor indicates that those effects are related to the yellow direction of the diagram. This result is seen in the PCA graphical analysis (**Figure 5A**).

Another interesting result can be observed in the graphical analysis of each single case (**Figure 5B**). Control subjects (labeled 1) were located in the right side of the factor 1 and were symmetrically spread around zero value of the factor 2. The deuteranomalous subjects (labeled 3) were more distant in factor 1, which means that they present stronger defects and also they were located in the positive values of factor 2 associated with the yellow projection, suggesting a high correlation between them.

## Discussion

In our paper, we showed consistent tritanopic color vision impairment in subjects with congenital color deficiency. Protanomalous and deuteranomalous subjects had higher thresholds of color discrimination compared with subjects with normal color vision. We were able to measure separated thresholds for the yellow and the blue regions of tritanopic axis. We also have strong information that supports the evidence that an abnormal L or M cone contributes to a statistically significant higher threshold on the yellow region.

Few studies were careful to analyze the yellow and blue regions of the tritanopic color confusion axis. In this sense, our paper corroborates previous studies showing separated channels for blue and yellow in which they reported longer reaction times to blue than green and yellow stimuli in normal color vision subjects (Medina and Diaz, 2006, 2010; Medina and Mullen, 2009). Additional evidence came from the study of Devos et al. (1996) in which the authors found bigger axis length in the MacAdam ellipses axis for blue–yellow discrimination in color congenital than normal subjects. Those results are in line with ours and could be considered an indirect result of our threshold measurements.

The main purpose of this research was to investigate possible tritanopic deficits in color discrimination of congenital color deficiency subjects and we were able to prove them. We had two main hypotheses about the tritanopic color discrimination impairment. The post-receptoral color vision mechanism is based on an opponency system of photoreceptors inputs to ganglion cells via bipolar cells. Two color pathways and one achromatic pathway leave the retina toward the higher subcortical and cortical processing levels. The Parvocellular pathway carries the color opponency of the L and M cones, and the Koniocellular pathway carries the S and (L + M cones; Cole et al., 1993; Dacey and Lee, 1994; Dacey, 1999; Packer et al., 2010). Since in congenital color deficiencies, the subjects express abnormal M or L pigments with a consequent shifting at the spectral absorption peak (Nathans et al., 1992; Deeb et al., 1994; Neitz and Neitz, 2000; Deeb, 2004, 2005; Neitz et al., 2004), it is quite reasonable to suppose that their color discrimination will be impaired for the colors in the tritanopic color confusion axis as well as for the evident red–green color confusion axes. However, the thresholds and scores found for most of the studies done on congenital color impairment subjects reveal that in the tritanopic color confusion axis, so far were found to be similar to those of normal color vision subjects (Pinckers et al., 1985; Birch, 1989; Ing et al., 1994; Kon and De, 1996; Hovis et al., 2004). The chromaticity discrimination in tritanopic color confusion axis in anomalous trichromats and dichromats subjects should impair their S cell’s discrimination as previously discussed. In our study, protanomalous and deuteranomalous subjects exhibit different color discrimination on tritanopic axis when compared to subjects with normal color vision which lead us to suggest that S cells had increased sensitivity to spectral shifts.

A recent study characterized the S+ and S- cells measured at the macaque LGN showed critical asymmetries between those S-cone cells (Tailby et al., 2008). The S- cells receive inputs from L and M cones over a larger region of retina, increasing the chances of L and M inputs having the same sign [S against (L + M)]. The S+ cells, however, receive inputs from smaller retina areas and given that the proportion of L cones is frequently higher than M cones, there is a higher chance that L and M cones inputs show opponent signs configuring the channel S- against L cells. Our results show that protanomalous subjects presented better color discrimination in the tritanopic color confusion axis than deuteranomalous subjects. We believe that S+ channel could support our findings. Considering the S+ channel of deuteranomalous subjects, the shift in the M cone spectral sensitivity closer to L cone should decrease the discrimination than the spectral sensitivity shift of L cone in direction to M cone presented in the protanomalous. This result supports the idea that deuteranomalous and protanomalous subjects had more complex perceptual differences for chromatic stimuli than we predicted.

We believe that the recent computerized method makes this kind of analysis easier to be implemented, due to its impressive methodological refinement. Chromaticity modifications are faster and reliable using computers and monitor screens. Computerized methods of study color discrimination for red–green and blue–yellow opponency, are far more superior to classical arrangement tests such as: FMH100, and pseudoisochromatic plates that have been reported in the literature (Ing et al., 1994; Birch, 1997; Ventura et al., 2003a,b, 2004, 2005a,b, 2007; Costa et al., 2005, 2006, 2007; Seshadri et al., 2005; Melamud et al., 2006; Paramei, 2012).

According to the PCA the increase of color discrimination thresholds at the tritanopic color confusion axis was due to the loss of discrimination generated by the yellow (or L + M cone) region of the axis. This is expected because the gene alteration in congenital color deficiency occurs in the chromosome that encodes both of those opsins (Nathans et al., 1992; Neitz and Neitz, 1995; Deeb, 2004, 2005). This idea is supported by the psychophysical findings in measurements of reaction time for chromatic stimulus that both red–green and blue–yellow post-receptoral channels had different mechanisms for each region (Medina and Diaz, 2006, 2010; Medina and Mullen, 2009). Nevertheless, our study found similar results using not only the hue dimension, but chromaticity coordinates; which are a more complex stimuli since it changes with hue and saturation simultaneously.

Asymmetries for thresholds measured by equiluminous spots for the blue, green, and yellow, but not the red sides of the color confusion axes were also found in subjects with optic nerve hypoplasia (Billock et al., 1994). This is an intriguing result and suggests a post-receptoral independence for each branch of the color opponent channel. The study of Devos et al. (1996) found that the thresholds along the red–green direction of the MacAdam ellipses minor axis (tritanopic axis) of the congenital color deficient subjects were significantly lower than those along the tritanopic axis measured for subjects with normal color vision. This result corroborates our findings suggesting additional tritanopic axis differences in congenital colorblindness. However, when the authors computed the tritanopic axis length (total distance between the ellipses borders) no difference was found leading them to conclude that there was no difference between the groups. Here, we have an important methodological point that could justify the absence of differences. The MacAdam ellipses adjustment was performed using the Least Squares Method which minimizes vast discrepancies by squaring the summed errors. In congenital color deficient subjects that method generates fitting distortions in the tritanopic color confusion axis in which the ellipses contour fitting frequently passes distant from the threshold measured. Additionally, our result shows a weighted contribution of the deuteranomalous for the yellow asymmetry. Hence, we suggest that the increase in the yellow region of the tritanopic color confusion axis is probably more related to the M than to the L cone contribution for the yellow color (L + M cone). Considering that our paper presents the first report on this matter, we consider that future works addressing these analyses in subjects with color congenital deficiencies are needed to confirm the hypothetical weighted M cone to yellow discrimination and to explore the L + M cone contributions in the tritanopic color discrimination reported in previous studies (Dacey and Lee, 1994; Dacey, 1996, 1999; Stromeyer et al., 1998; Horwitz et al., 2005; Diaconu and Faubert, 2006; Packer et al., 2010).

## Conclusion

The paper emphasizing our main findings: that the tritanopic color confusion axis impairment is present in subjects with congenital color deficiencies. The yellow region of the tritanopic color confusion axis was more evidently affected when compared with the blue region. Additionally, we found that deuteranomalous subjects were more affected than the protanomalous subjects; suggesting different and more complex color discrimination performances between them that we traditionally expect.

## Author Contributions

All authors listed, have made substantial intellectual contribution to the work, and approved it for publication. MC performed all statistical analysis, figures and wrote the first versions of the MS. PG collected almost of the CCT data. MB and DV performed substantial modifications in the MS and also collected some data.

## Conflict of Interest Statement

The authors declare that the research was conducted in the absence of any commercial or financial relationships that could be construed as a potential conflict of interest.
